# Ultrasound-induced modifications of *Auricularia auricula* polysaccharides: Implications for antioxidant and glucose-lowering activities

**DOI:** 10.1016/j.fochx.2025.102209

**Published:** 2025-01-25

**Authors:** Te Yu, Yonggang Dai, Zifei Wang, Yang Gao, Qiong Wu

**Affiliations:** a Department of Food Science and Engineering, College of Food Science and Engineering, Changchun University, No.6543, Satellite Road, Changchun 130022, China; b Jilin Academy of Agricultural Sciences, Changchun, Jilin Province 130033, China

**Keywords:** *Auricularia auricula* polysaccharides, Ultrasonic degradation, Antioxidant activity, Hypoglycemic activity, Hypolipidemic activity, Functional food

## Abstract

High molecular mass and poor solubility of *Auricularia auricula* polysaccharides (AAP) limit their physiological activity. This study employed ultrasonic degradation to address these challenges. Ultrasonic treatment reduced the molecular mass of AAP from 1 × 10^5^ Da to 3.8 × 10^3^–8 × 10^4^ Da, enhanced hydroxyl group exposure, and significantly improved solubility, with dissolution time reduced to one-ninth of the pre-degradation level at 20 °C. The antioxidant activities of AAP were enhanced after ultrasonic degradation, with DPPH scavenging IC_50_ reduced from 3.58 mg/mL to 2.07 mg/mL and hydroxyl radical IC_50_ from 2.28 mg/mL to 1.77 mg/mL. Hypoglycemic effects were improved, with α-amylase and α-glucosidase inhibition rates increasing by 5.06 % and 5.81 %, respectively. Bile acid binding for MBS improved by 28.6 %, indicating ultrasonic degradation enhanced hypolipidemic activity. This study demonstrates ultrasonic degradation is a green, efficient method to improve AAP's functional properties, which broadens its applications in functional foods and health supplements.

## Highlights

• Ultrasonic degradation alters the structure and enhances the bioactivity of Auricularia auricula polysaccharides
• Ultrasonic degradation reduces the molecular mass of Auricularia auricula polysaccharides.
• Ultrasonic degradation improves solubility and physicochemical properties of Auricularia auricula polysaccharides.

## 1 Introduction

*Auricularia auricula* polysaccharides (AAP), extracted from the fruiting bodies of *Auricularia auricula*, are the primary bioactive components of this traditional edible and medicinal fungus (Yu et al., 2023a). AAP is well recognized for its health-promoting properties, including antioxidant, hypoglycemic, antitumor, and immunomodulatory effects (Dong et al., 2024). Despite these promising biological activities, the application of AAP still faces significant challenges due to its high molecular mass and complex chemical structure. The intricate structure of AAP, characterized by diverse branching and high molecular mass, enhances their biological activities but also makes them difficult to study and utilize effectively. Additionally, the high viscosity of AAP solutions further complicates their use, as it limits the ability of the polysaccharides to cross multiple cellular barriers, reducing their bioavailability and efficacy in the human body (Wang, Ma, et al., 2024).

To overcome these challenges, numerous degradation methods have been devised to improve the viscosity of AAP. For example, Ma et al. employed a solution plasma process (SPP) with hydrogen peroxide (H_2_O_2_) to degrade AAP, demonstrating that the degraded polysaccharides exhibited enhanced antioxidant activity and reduced viscosity. However, their study did not explicitly evaluate the impact of degradation on the bioavailability of AAP, which remains an area requiring further investigation (Ma et al., 2018). While chemical methods like SPP are effective, they face limitations in food industry applications due to safety concerns associated with the use of organic reagents. Enzymatic degradation, such as the use of mannanase by Huang et al., has also demonstrated improved antioxidant activity of AAP. However, enzymatic methods often require precise conditions, including controlled pH, temperature, and enzyme specificity, making them less suitable for large-scale or complex food processing environments. These limitations highlight the need for alternative, efficient, and industry-friendly degradation techniques (Huang et al., 2022).

Compared to chemical and biological methods, physical degradation techniques offer several advantages, including environmental friendliness, high efficiency, ease of operation, and better preservation of polysaccharide activity. Among these, ultrasonic degradation stands out as an effective approach. Ultrasonic degradation has advantages over microwave treatment, such as concentrated energy, more uniform heating of the reaction system, and a gentler degradation process (Wang et al., 2024). The mechanism by which ultrasound affects the properties of polysaccharides can be summarized as follows: Ultrasound generates cavitation effects that rupture cell walls and accelerate mass transfer, influencing the molecular weight, monosaccharide composition, and structural characteristics of polysaccharides. While ultrasound treatment generally does not alter the primary chemical structure of polysaccharides, it can reduce molecular weight and modify monosaccharide ratios, potentially enhancing their antioxidant activity. Moreover, the mechanical shear and thermal effects of ultrasound can improve the solubility and physiological activity of polysaccharides. Qiu et al. demonstrated that ultrasonic treatment reduced the viscosity and molecular mass of AAP. However, their study focused on limited physicochemical properties. In contrast, we further explore the hypoglycemic and hypolipidemic activities of ultrasonically degraded AAP, providing insights into its broader functional applications and structural changes (Qiu et al., 2019).

In our previous work (Yu et al., 2023b), we utilized AAP to create biofilms for beef preservation and observed that the initially poor solubility of AAP was significantly improved by ultrasonic treatment. This improvement was primarily attributed to the cavitation effect during ultrasonication, where collapsing microbubbles generated intense shear forces and localized high pressure, broke glycosidic bonds and reduced molecular mass. These changes prompted us to conduct a more comprehensive and systematic study (Yu et al., 2023b). In this study, ultrasonic degradation techniques were employed to systematically investigate the effects on the structural characteristics, antioxidant, hypoglycemic, and hypolipidemic activities of AAP. The aim was to enhance the bioavailability and functional properties of AAP, thereby improving its potential applications in functional foods and health supplements. However, a limitation of this study is that the specific mechanisms underlying the regulatory effects of ultrasonically degraded AAP were not explored, which could be addressed in future research to provide a deeper understanding of its functional properties.

## 2 Materials and methods

### 2.1 Materials

*Auricularia auricula* were obtained from Walmart Inc. (Changchun, China). The selection criteria for *auricularia auricula* included factors such as the freshness of the raw *Auricularia auricula* material, the absence of visible impurities, and compliance with food-grade quality standards. The raw materials were further processed to ensure uniformity and suitability for subsequent analysis. Analytical grade acetonitrile was supplied by Shanghai Macklin Biochemical Co., Ltd.; methanol, anhydrous ethanol, ascorbic acid, hydrochloric acid, hydrogen peroxide, ferrous sulfate, trichloroacetic acid were obtained from Beijing Chemical Works; resorcinol, tris (hydroxymethyl) aminomethane, salicylic acid, potassium ferricyanide, ferric chloride, 1,1-diphenyl-2-picrylhydrazyl (DPPH), acarbose, α-amylase, α-glucosidase, sodium deoxycholate, taurocholate, and Glycocholate was obtained from Shanghai Aladdin Biochemical Technology Co., Ltd., and all reagents used in this study were of analytical grade.

### 2.2 Preparation of crude AAP

Crude AAP was extracted using a water extraction and alcohol precipitation method, achieving a yield of 16.96 %. A total of 500 g of dried *Auricularia auricula* powder (passed through a 20-mesh sieve) was mixed with 80 % ethanol at a material-to-liquid ratio of 1:10 (g/mL), and extracted twice at 70 °C for 2 h each time. The mixture was filtered and the residue was dried. The dried residue was then extracted twice with distilled water at a material-to-liquid ratio of 1:30 (g/mL) at 90 °C for 4 h each time. After extraction, the mixture was centrifuged at 3500 r/min for 20 min to remove the precipitate, and the supernatant was concentrated by rotary evaporation to one-fourth of its original volume to obtain a crude polysaccharide solution. Trichloroacetic acid was added to the crude solution for deproteinization, and the mixture was kept at 4 °C for 24 h. It was then centrifuged at 4000 r/min for 20 min to collect the supernatant. An equal volume of anhydrous ethanol was added to precipitate the crude polysaccharides, and the mixture was kept at 4 °C for another 24 h before being centrifuged at 8000 r/min for 20 min. The crude polysaccharides were obtained by freeze-drying. Pigments were removed using AB-8 macroporous resin column chromatography, selected for its high adsorption efficiency, chemical stability, and suitability for separating polysaccharides, with a flow rate of 1.5 mL/h^9^.

### 2.3 Isolation and purification of AAP

Neutral acidic AAP elution using DEAE-52 cellulose was performed as follows: 100 g of cellulose was taken and soaked using distilled water for one week to make the cellulose soak completely, and then soaked in 2 L of 0.5 mol/L NaOH solution for 1 h. The cellulose was initially washed with distilled water until neutralization, followed by treatment in 2 L of 0.5 mol/L HCl solution for 1 h. Subsequently, the cellulose was thoroughly rinsed with distilled water to remove any residual acid, and vacuum filtration was employed to eliminate air bubbles. The column was loaded, and the mobile phase flow rate was fixed at 3 mL/h. The column was rinsed with 2 times the volume of distilled water, then rinsed with 2 times the volume of 2 mol/L sodium chloride solution, and then rinsed with distilled water again, and silver nitrate was used to determine whether it was cleaned. 4 g of AAP were dissolved in 150 mL of distilled water and filtered through a 0.45 μm membrane to remove insoluble matter. The samples were first eluted with distilled water to remove neutral sugars, and fractions were collected using an automatic collector. The phenol‑sulfuric acid method was employed to confirm the removal of neutral sugars. The saturated NaCl solution was mixed with water using a gradient mixer to elute the acidic AAP, which was then collected by an automatic collector. The elution curve was used to determine the content of AAPs based on the phenol‑sulfuric acid method. The acidic AAP was obtained by elution using the best elution concentration of the elution curve. 250 mg of the polysaccharide sample was dissolved in 5 mL of water, the precipitate was removed using high-speed centrifugation, and the supernatant was chromatographed on a Sephadex LH-20 gel chromatography column. The column was eluted with 0.15 mol/L NaCl solution at a flow rate of 0.15 mL/min, and 3 mL of eluate was collected each time. The phenol‑sulfuric acid method was used to detect the sugar content in the eluate, and the main polysaccharide fractions were collected. The final refined AAP was processed by dialysis and concentration, followed by lyophilization (Wang & Guo, 2020).

### 2.4 Ultrasonic degradation

The method was adapted from Qiu et al (Chen et al., 2024), with modifications for improvement. Aqueous solutions of AAP, obtained from isolation and purification, were prepared with pH levels adjusted to (5, 6, 7, 8, and 9) using hydrochloric acid and sodium hydroxide. Material-to-liquid ratios were adjusted to (1:10, 1:20, 1:30, 1:40, and 1:50) (mg/mL). Each 10 mL aliquot was treated using an ultrasonic cell disrupter, with the temperature controlled by an ice bath during sonication. The degradation was performed under varying conditions, including sonication times (1, 2, 3, 4, and 5 h) and sonication powers (600, 700, 800, 900, and 1000 W). Post-sonication, the degraded solutions were freeze-dried to yield modified AAP.

### 2.5 Molecular mass measurement

The separation was carried out on a TSK-gel G-4000 PWXL stainless steel column (7.8 × 300 mm) with a RID-10 A refractive index detector at a column temperature of 35 °C, and the mobile phase was ultrapure water at a flow rate of 0.5 mL/min. A molecular mass standard curve was drawn using the retention time as the horizontal coordinate, and the logarithm of the molecular mass of the standard dextran (logMw) as the vertical coordinate. According to the standard curve, the molecular mass of AAP was calculated (Xi et al., 2024).

### 2.6 Determination of monosaccharide composition

A high-performance liquid chromatography (HPLC) system of LC-10AVD from Shimadzu, Japan was used, with an SPD-10AVD ultra*v*iolet detector, a DIKMAInertsilODS-3 column (4.6 × 150 mm), and the mobile phase was a mixture of PBS (0.1 mol/L, pH 70) and acetonitrile in the ratio of 82:18 *v*/v at a flow rate of 1.0 The flow rate was 1.0 mL/min and the detection wavelength was 245 nm^13^.

### 2.7 Scanning electron microscope (SEM)

The microstructures of dialyzed AAP samples obtained through different degradation conditions were observed using field emission scanning electron microscopy, respectively. The specific methods were as follows: The AAP was fixed onto the specimen holder with conductive adhesive and coated with gold for 30 s. Perform SEM (S-4800 SEM, Hitachi Ltd., Tokyo, Japan) on the sample under vacuum conditions with a voltage of 30 kV and a working distance of 1–5 mm. Inspect the microstructure of the sample at room temperature (Meng et al., 2021).

### 2.8 Fourier transform infrared (FTIR) spectroscopy

The bonding and functional groups of the degraded AAPs were analyzed by FTIR with an IS50 spectrometer (SuZhou AOPS Plasma Technology Co., Ltd.). The degraded AAP sample was dried at 105 °C for 24 h. Then, 2 mg of the dried sample was thoroughly mixed with 200 mg of spectroscopic-grade potassium bromide. This mixture was pressed into a transparent and uniform disk under a force of 80KN. The disk was then scanned using FTIR in the wavelength range of 4000 to 400 cm^−1^at a resolution of 4 cm^−1^. The analysis was performed with 32 scans to ensure accurate spectral data collection. The corresponding IR spectral data were recorded and used to identify the bonding and functional groups of the degraded AAPs (Yang et al., 2023).

### 2.9 Nuclear magnetic resonance (NMR)

15 mg of polysaccharides before and after degraded AAP were weighed separately and fully dissolved in 0.5 mL of D2O. The ^1^H NMR, ^13^C NMR, and HSQC spectra of the samples were detected by superconducting nuclear magnetic resonance (NMR) spectrometer AVANCE 300, with ^1^H NMR detected at 600 MHz and ^13^C NMR detected at 150 MHz^16^.

### 2.10 Solubility determination

Samples of 0.1 g AAP, both before and after degradation, were weighed and dissolved in 100 mL of distilled water. The solutions were magnetically stirred at various temperatures (20, 40, 60, 80, and 100 °C) until fully dissolved, and the dissolution time was recorded from the start of stirring to complete dissolution (Postulkova et al., 2017). Each sample was measured in triplicate.

### 2.11 Determination of antioxidant activity

#### 2.11.1 DPPH rree radical scavenging rate

A certain amount of DPPH was weighed and prepared into 0.1 mmol/L DPPH solution with anhydrous ethanol. Separately, 40 μL of degraded AAP solution was taken and 160 μL of DPPH solution was added, mixed well, and left at room temperature for 30 min, then centrifuged at 5000 r/min for 10 min. The absorbance value of the supernatant was measured at 517 nm, and the experiment was repeated three times (Luo et al., 2025). The scavenging rate of samples against DPPH radicals was calculated by the following formula:

(1) $$\text{DPPH Free radical scavenging rate}\left(\%\right)=\left(1-\frac{A_{1}-A_{2}}{A_{0}}\right)\times 100\%$$

A_0_ in the formula is the absorbance of 40 μL of water +160 μL of DPPH solution, A_1_ is 40 μL of sample + 160 μL of DPPH solution, and A_2_ is 40 μL of sample + 160 μL of anhydrous ethanol.

#### 2.11.2 Hydroxide radical scavenging rate

The hydroxyl radical scavenging capacity was determined according to the method of Lee et al. To a 15 mL centrifuge tube, 2 mL of ferrous sulfate solution (6 mmol/L), 2 mL of hydrogen peroxide solution (6 mmol/L), 2 mL of salicylic acid solution (6 mmol/L), and 2 mL of degraded AAP solution were added, and the reaction was carried out at 37 °C for 1 h. The absorbance of the solution was measured at 517 nm using distilled water as a reference and the experiments were repeated three times (Lee et al., 2023). The hydroxyl radical scavenging rate was calculated according to the following formula:

(2) $$\text{Hydroxyl radical scavenging rate}\left(\%\right)=\left(1-\frac{A_{1}-A_{2}}{A_{0}}\right)\times 100\%$$

In the formula: A_0_ is the absorbance of 2 mL ferrous sulfate solution +2 mL hydrogen peroxide solution +2 mL salicylic acid +2 mL distilled water; A_1_ is the absorbance of 2 mL ferrous sulfate solution +2 mL hydrogen peroxide solution +2 mL salicylic acid +2 mL sample solution; A_2_ is the absorbance of 2 mL ferrous sulfate solution +2 mL distilled water +2 mL salicylic acid +2 mL sample solution.

#### 2.11.3 Superoxide anion scavenging rate

To the 96-well plate, 50 μL of sample, 50 μL of 300 μmol/L NBT solution, 50 μL of 936 μmol/L NADH solution, and 50 μL of 120 μmol/L PMS solution were added, and the reaction was mixed and protected from light for 30 min^20^. Each sample was measured in triplicate. The specific equations were as follows:

(3) $$\text{Superoxide anion scavenging rate}=\left(1-\frac{A_{1}-A_{2}}{A_{0}}\right)\times 100\%$$

In the formula, the absorbance value was measured at 570 nm, which was recorded as A_1_, and the absorbance value was recorded as A_2_ when distilled water was used instead of PMS solution, and A_0_ when polysaccharide samples were used instead.

### 2.12 Evaluation of hypoglycemic activity in vitro

#### 2.12.1 α-amylase inhibitory activity

The inhibitory activity of α-amylase was determined according to the method of Deng et al (Deng et al., 2020). In brief, AAP solutions with varying concentrations (1.0–5.0 mg/mL), NaCl solution (6 mM), and DNS reagent were prepared in 0.1 M phosphate buffer (PBS, pH 6.8). The α-amylase enzyme was dissolved in 6 mM NaCl solution at a concentration of 0.5 mg/mL. The DNS reagent, consisting of 120 mg/mL sodium potassium tartrate and 10 mg/mL 3,5-dinitrosalicylic acid, was prepared in 0.4 M sodium hydroxide. For the assay, 0.5 mL of AAP solution was mixed with 0.5 mL of α-amylase solution, and the mixture was incubated at 37 °C for 10 min. The reaction continued after the addition of 0.5 mL of AAP solution, with further incubation at 37 °C for 15 min. To terminate the reaction, 1 mL of DNS reagent was added, and the mixture was heated in boiling water for 10 min. After cooling to room temperature, the absorbance of the resultant solution was measured at 540 nm. Each sample was measured in triplicate. PBS was used as the blank control, and acarbose served as the positive control:

(4) $$\alpha -\text{amylase inhibition rate}=\left(1-\frac{A_{s}-A_{0}}{A_{b}}\right)\times 100\%$$

where A_s_ was the absorbance of the sample, A_b_ was the absorbance of PBS, and A_0_ was the absorbance of the sample without α-amylase.

#### 2.12.2 α-glucosidase inhibitory activity

A volume of 0.3 mL of AAP solution at various concentrations was pipetted into test tubes, with acarbose used as a positive control. Subsequently, 1.2 mL of PBS and 0.25 mL of α-glucosidase solution were added to each tube, followed by incubation in a water bath at 37 °C for 15 min. Afterward, 0.25 mL of p-nitrophenol glucopyranoside solution was added, and the reaction was allowed to proceed at 37 °C for an additional 25 min. Each sample was measured in triplicate. To terminate the reaction, 1 mL of sodium carbonate solution was added (Deng et al., 2020). The inhibition rate was calculated by the following formula:

(5) $$\alpha -\text{glucosidase inhibition rate}=\left(1-\frac{A_{0}-\left(A_{1}-A_{2}\right)}{A_{0}}\right)\times 100\%$$

In the formula: cooled to room temperature, the absorbance value was measured at 405 nm A_1_. The enzyme solution was replaced with water and measured A_2_. The sample solution was replaced with water as a blank control A_0_.

### 2.13 Evaluation of hypolipidemic activity in vitro

The aqueous solution of AAP at concentrations of 2, 4, 6, 8, and 10 mg/mL (1 mL each) before and after degradation was prepared in a stoppered test tube. To each test tube, 1 mL of artificial gastric fluid was added, followed by oscillation digestion at 37 °C constant temperature for 1 h. The pH value was then adjusted to 6.8, and 4 mL of artificial intestinal fluid was added. After oscillation digestion at 37 °C constant temperature for 1 h, 4 mL of 1.0 mmol / L bile salt solution was introduced, and the mixture oscillated at 37 °C constant temperature for an additional 1 h. The samples were then centrifuged at 4000 r/min for 20 min, and 2.5 mL of supernatant was collected. To this, 7.5 mL of 60 % sulfuric acid solution was added and mixed thoroughly. Following another centrifugation at 4000 r/min for 20 min, 2.5 mL of supernatant was again taken, to which 7.5 mL of 60 % sulfuric acid solution was added and mixed well. The solution was then heated in a water bath at 70 °C for 20 min, immediately followed by cooling in an ice bath for 5 min. Absorbance was measured at the wavelength of 387 nm^22^. Each sample was measured in triplicate. Based on the corresponding standard curve, the bile acid salt content in the supernatant was calculated as the unbound bile acid salt content using the following formula:

(6) $$\text{Bile acid salt binding rate}=\frac{A_{0}-A_{1}}{A_{0}}\times 100\%$$

In the formula: A_0_: is the amount of bile acid salt added; A_1_: is the remaining amount of bile acid salt.

### 2.14 Statistical analysis

All experiments were conducted in multiple replicates, with the data expressed as mean ± standard deviation. Statistical analysis was done using SPSS, and the figures were created with Origin software.

## 3 Results and discussion

### 3.1 Molecular mass comparison

**Fig. 1:**
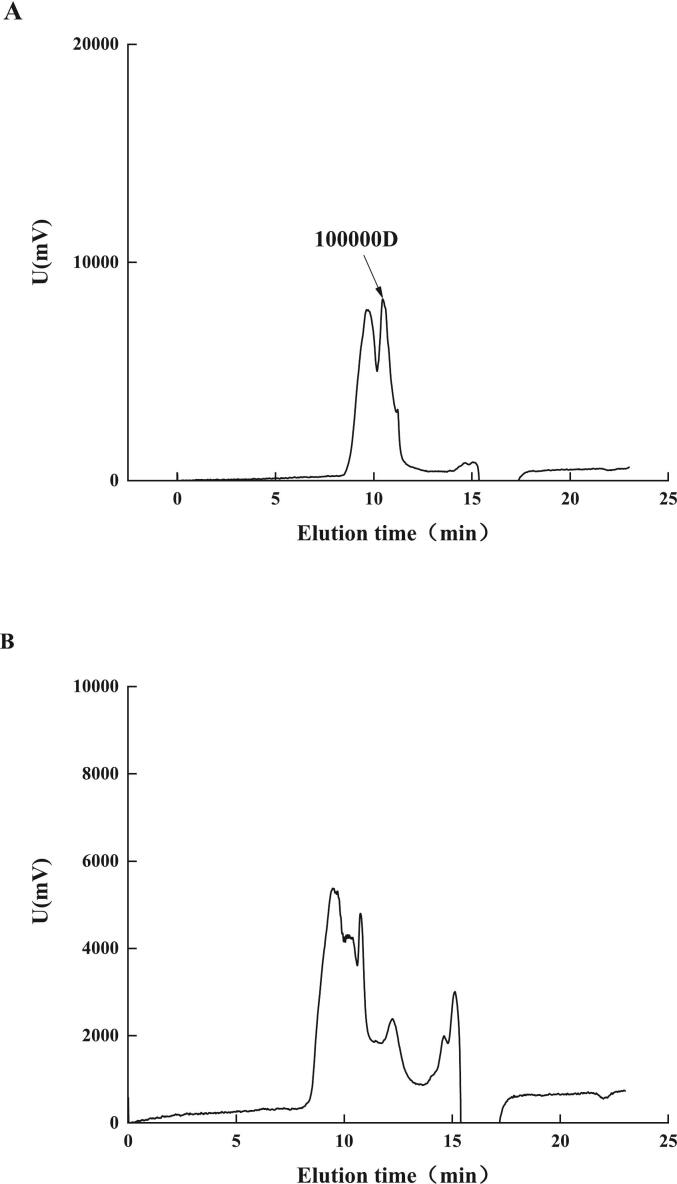
Molecular mass-distribution of AAP before (A) and after (B) ultrasonic degradation.

From Fig. 1, comparing the molecular mass distribution of AAP before and after degradation, it can be seen that the molecular mass of AAP before degradation is above 1 × 10^5^ Da, but after ultrasonic degradation, mainly concentrated in 5 × 10^4^ Da - 8 × 10^4^ Da molecular mass, and the lowest can reach 3.804 × 10^3^ Da. This indicates that ultrasonic waves play a more obvious degradation effect on AAP. The broader molecular mass distribution observed after degradation refers to the presence of AAP fragments with a wider range of molecular weights. This indicates that ultrasonic treatment breaks down AAP into fragments of varying sizes, potentially enhancing their biological activities by exposing more active sites (Huang et al., 2018). Additionally, a lower molecular mass is often associated with improved solubility and bioavailability, which can enhance the application potential of AAP in functional foods and pharmaceuticals (Spann & Boucher, 2023). The significant reduction in molecular mass also implies that ultrasonic degradation can be effectively controlled to obtain AAP with desired molecular characteristics, offering versatility for different applications. This reduction can be attributed to the physical effects of ultrasonic cavitation. Cavitation generates high-energy microbubbles that collapse rapidly, creating localized high pressure, shear forces, and elevated temperatures. These intense forces disrupt the glycosidic bonds within the polysaccharide chains, resulting in the cleavage of long-chain polysaccharides into smaller fragments. The exposure of hydroxyl groups and other functional groups on the polysaccharide backbone further supports this molecular breakdown. In addition to reducing molecular mass, ultrasonic degradation enhances the solubility of AAP, likely due to the smaller molecular size and increased surface area, which facilitates better interaction with water molecules (Wang et al., 2023a). Furthermore, the molecular mass-reduction enhances the permeability of polysaccharides, which could be beneficial for absorption in the gastrointestinal tract, thus increasing their potential efficacy as bioactive components. The wide range of molecular mass after degradation also suggests that ultrasound treatment can be optimized for specific applications, where certain molecular mass ranges are more effective for particular biological functions.

### 3.2 Microstructure

**Fig. 2:**
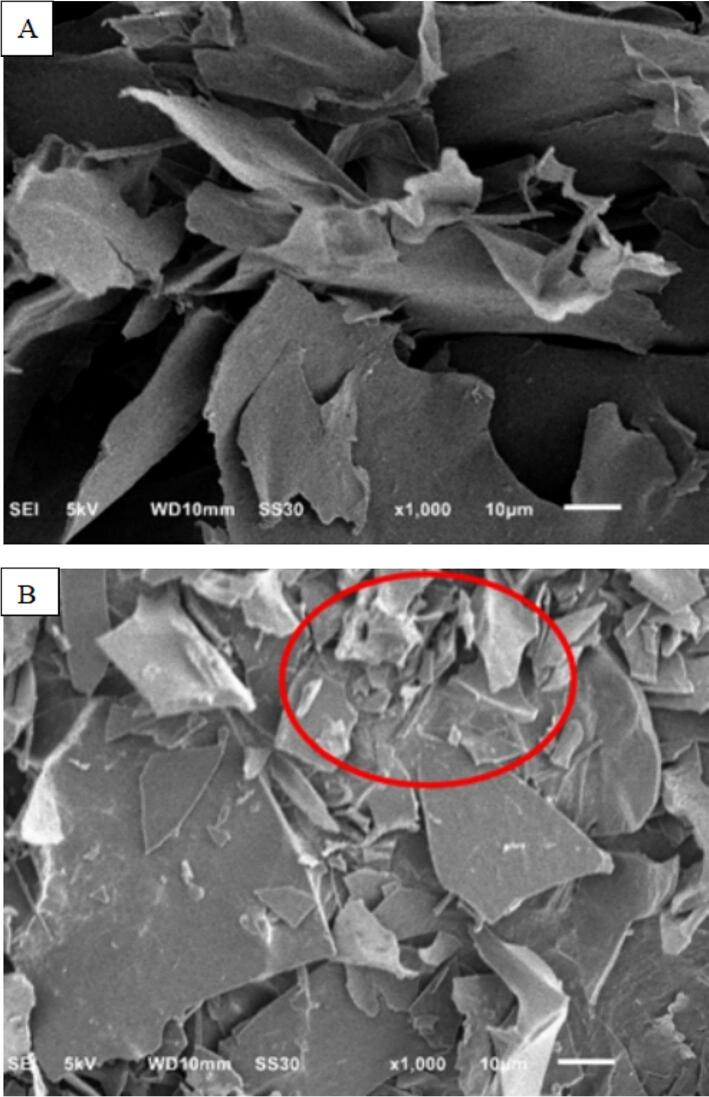
Comparison of electron microscopy images of AAP before (A) and after (B) ultrasonic degradation.

From the scanning electron microscope Fig. 2, it can be seen that the fragmentation structure of black wood polysaccharide increased significantly after ultrasonication, and the surface of solid aggregates of polysaccharides was arranged more orderly, which may be due to the destruction of covalent bonds in the molecules of AAP after ultrasonication, and the breakage of molecular bonding sites was regular, and the polysaccharide particles appeared to be agglomerated and adhered to the phenomenon so that the surface of polysaccharides solid agglomerates became flat and orderly. The increased fragmentation and orderly arrangement observed in the microstructure suggest that ultrasonic treatment causes significant physical changes to AAP. These changes can lead to enhanced surface area, which may improve the interaction between AAP and other molecules, such as enzymes or bioactive compounds, thereby potentially enhancing its biological activity. The reduction in particle size and the more compact, orderly structure may also facilitate better dispersion in aqueous systems, which is advantageous for its use in food formulations. The destruction of covalent bonds and subsequent rearrangement of the polysaccharide structure can enhance the overall stability of AAP in different environmental conditions, making it more suitable for various applications in functional foods and nutraceuticals (Xiong et al., 2019).

### 3.3 Monosaccharide composition

The results of determining the monosaccharide composition of AAP before and after ultrasonic degradation are shown in Table 1, and it was found that ultrasound had no significant effect on the monosaccharide composition of AAP, but the molar ratio would be different. Glucuronic acid content remained the most abundant after ultrasonic degradation with a small increase. The slight change in the molar ratio of monosaccharides indicates that while ultrasonic treatment affects the molecular mass and physical structure of AAP, it does not significantly alter its fundamental chemical composition. This preservation of monosaccharide composition is crucial for maintaining the inherent bioactive properties of AAP. The small increase in glucuronic acid content could enhance the anionic character of AAP, which might improve its ability to interact with positively charged molecules, such as certain minerals or proteins, thus broadening its functional applications (He et al., 2022). The consistency in monosaccharide composition also suggests that ultrasonic treatment is a mild method that can be used to modify polysaccharides without compromising their chemical integrity.

**Table 1:** Monosaccharide composition of AAP before and after ultrasonic degradation.

	Mannose	D-Glucuronic acid	Galacturonic acid	Xylose	Arabinose	Fucose
AAP before degradation	14.77 %	48.25 %	19.25 %	9.57 %	4.66 %	3.46 %
AAP after degradation	13.62 %	49.47 %	18.34 %	10.38 %	4.53 %	3.62 %

### 3.4 Infrared spectral analysis

**Fig. 3:**
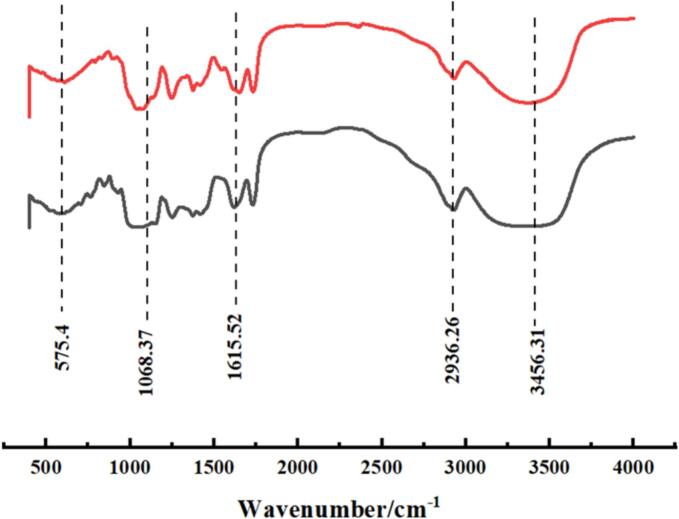
Infrared spectrum analysis of AAP before and after degradation.

The IR spectra of AAP before and after degradation are shown in Fig. 3. where the post-degradation sample is represented by the red curve and the pre-degradation sample is represented by the black curve. The results showed that although both samples showed absorption peaks at 1060 cm^−1^ and 1615 cm^−1^, which corresponded to the stretching vibration of the C-OH bond in sugar molecules, the absorption peaks of the post-degraded AAP were sharper and more intense than those of the pre-degraded sample (Sample B) at these wave numbers. Moreover, in the high wavenumber region, especially at 2935 cm^−1^ and 3450 cm^−1^, both samples exhibited absorption peaks attributed to the stretching vibrations of -CH and O—H bonds, respectively. Notably, the peaks of the degraded samples were more pronounced at these wave numbers, especially the strong absorption peak at 3450 cm^−1^, indicating the presence of more hydroxyl functional groups (Ren & Liu, 2020). The increase in intensity of the absorption peaks after ultrasonic treatment suggests that more functional groups, particularly hydroxyl groups, were exposed as a result of the degradation process. This increased exposure of hydroxyl groups is likely responsible for the enhanced antioxidant activity observed in the degraded AAP, as these groups can donate hydrogen atoms to neutralize free radicals (Gong et al., 2017). The sharper peaks also indicate a more uniform structure, which may contribute to improved functional properties, such as solubility and reactivity. The pronounced absorption at 3450 cm^−1^ implies that ultrasonic treatment enhances the hydrophilic nature of AAP, which could be beneficial for its incorporation into aqueous food systems or for enhancing its interaction with other hydrophilic compounds (Wang et al., 2016).

### 3.5 Nuclear magnetic resonance measurements

**Fig. 4:**
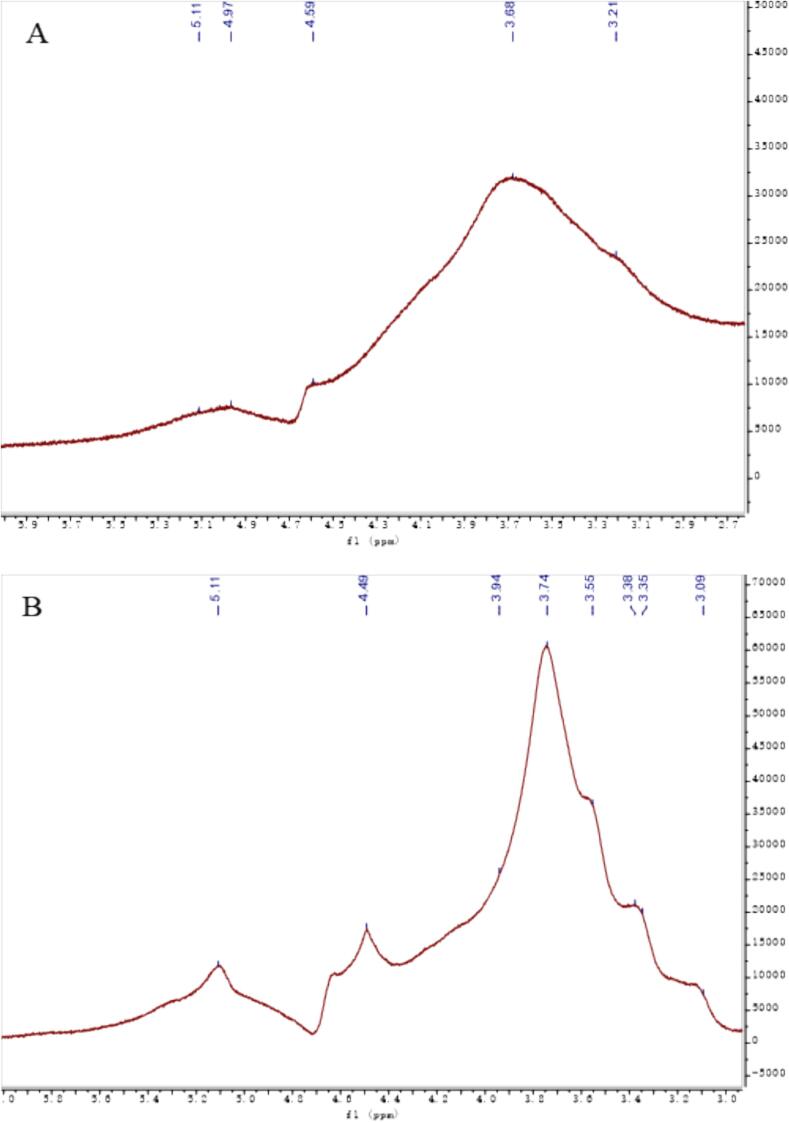
Hydrogen spectrum before (A) and after (B) ultrasonic degradation.

**Fig. 5:**
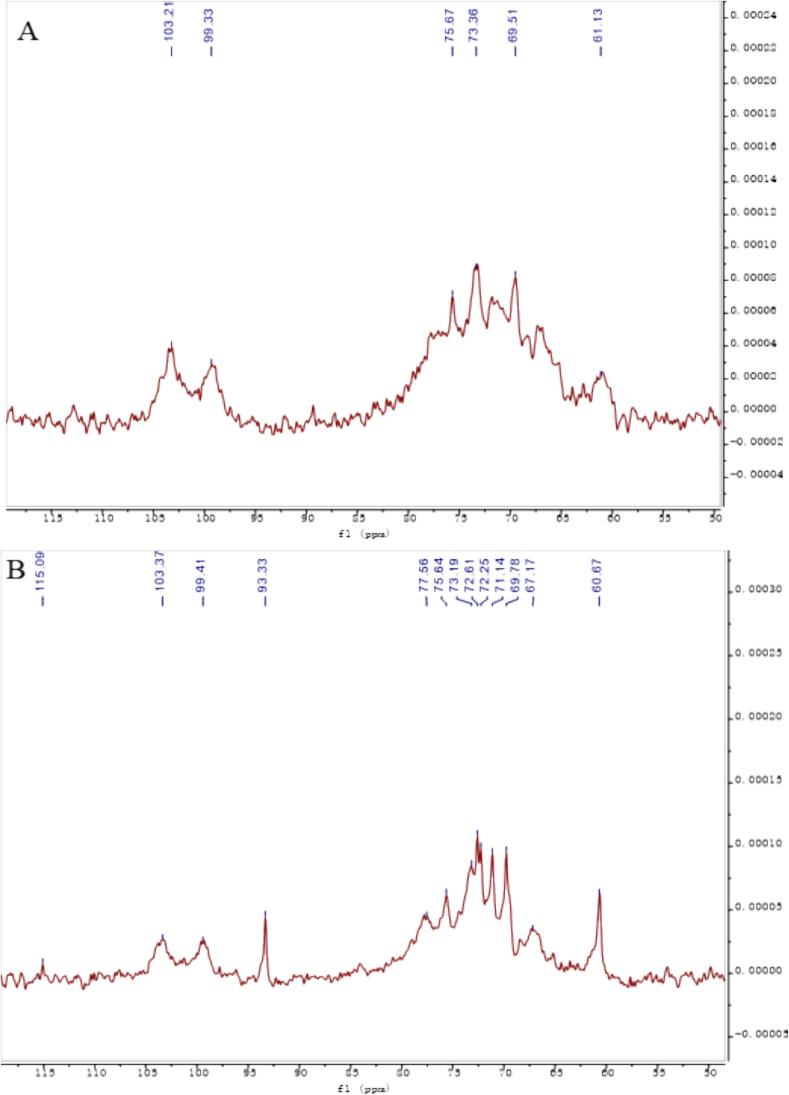
Carbon spectrum before (A) and after (B) ultrasonic degradation.

**Fig. 6:**
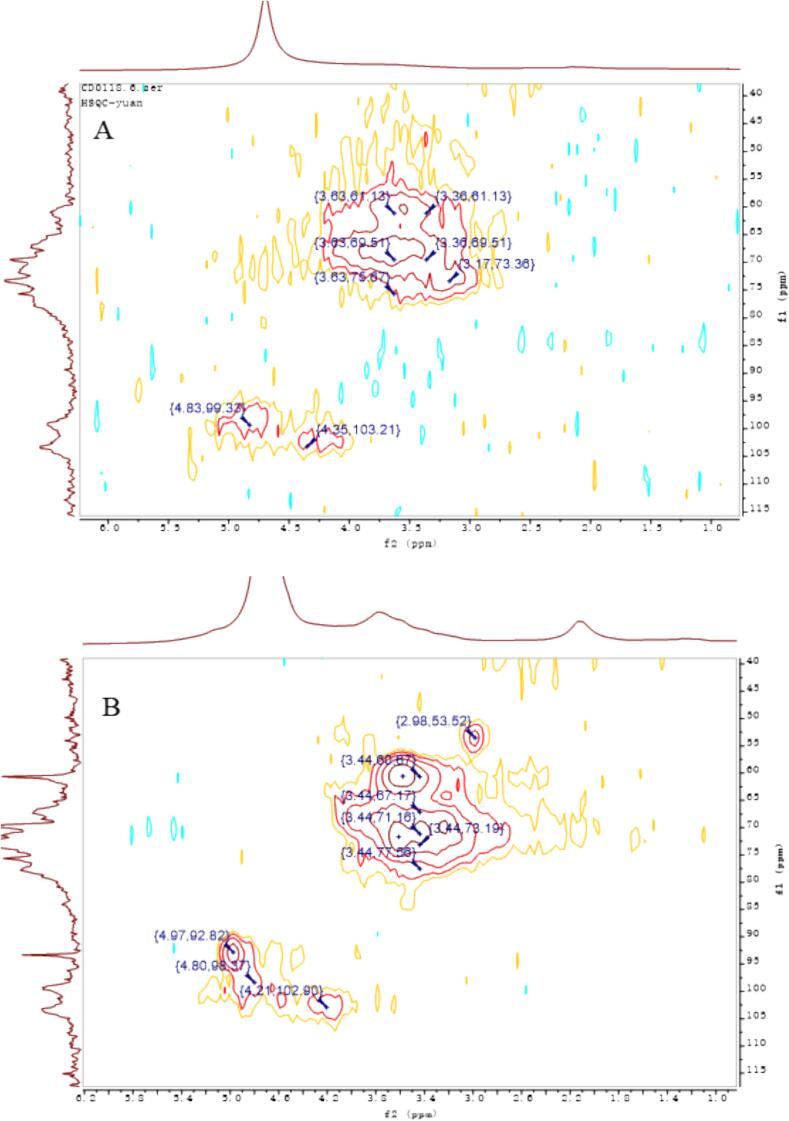
HSQC diagram before (A) and after (B) ultrasonic degradation.

**Fig. 7:**
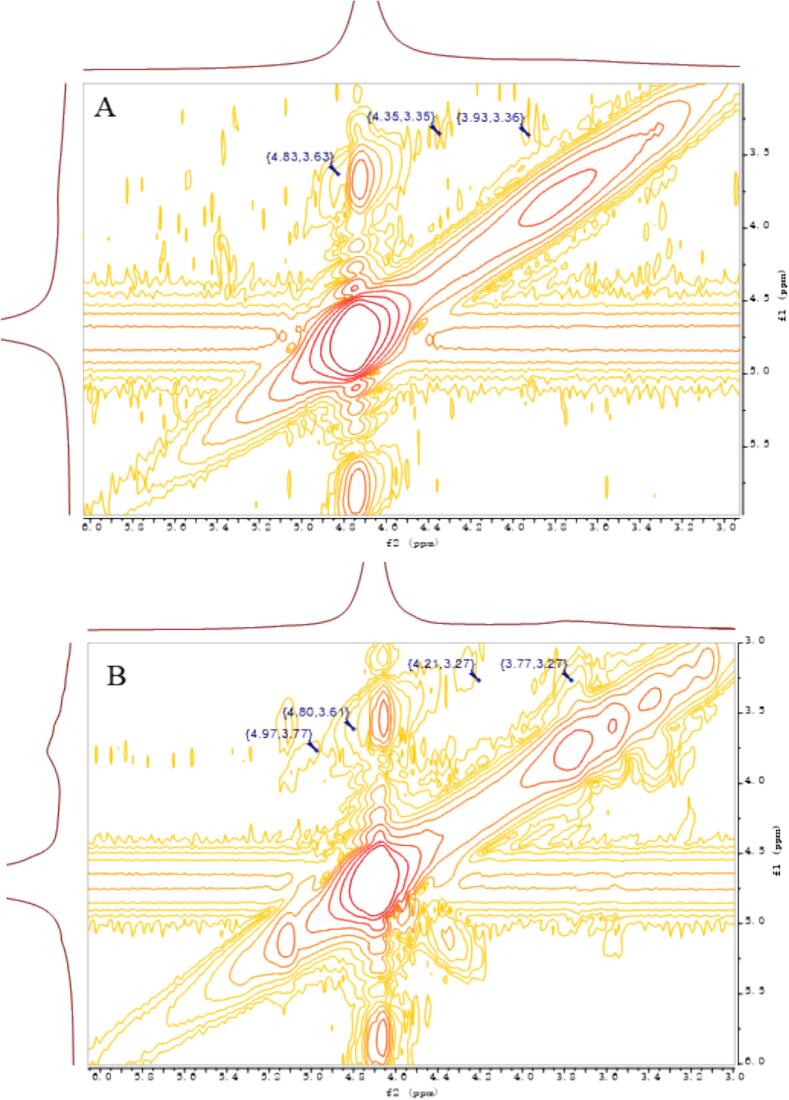
COSY diagram before (A) and after (B) ultrasonic degradation.

For the extracted AAP, structural changes before and after sonication were inferred from the following key NMR spectra. The NMR spectra in Fig. 4 became clearer after ultrasonic treatment. This indicates improved solubility and better signal clarity. The peak out in the heterohead region was weak and it was difficult to judge the detailed changes before and after ultrasonic degradation, so the following multiple spectra were used to analyze and verify the structural changes. As can be seen from the NMR carbon spectra before and after sonication in Fig. 5, the hetero-head position corresponds to 99.33 for the hetero-head C_1_ signal of the main chain α-1,3-Man and 103.21 for the hetero-head C_1_ signal of the branched β-1,6-Glc (Jiang et al., 2023). What can be seen in the figure is that there is a more obvious change in the hetero-head region (90–110 ppm), in which the two original hetero-head regions at 103 and 99 ppm still exist, confirming that the 1,3-Man and 1,6-Glc glycosidic bonds constituting the backbone of the structure are still present in large quantities and that the β-1,6-Glc glycosidic bond at 103 ppm has become relatively weaker, confirming that a part of it is undergoing reaction. It is assumed that the glycosidic bond was broken to form a new C_1_-reducing end during the sonication, and the newly generated carbon signal at 93 ppm corresponds to the C_1_ of this reducing end. By comparing the before and after changes, it can be initially judged that the branched chain of this mannan has undergone partial glycosidic bond breakage, resulting in a decrease in the average molecular mass. As can be seen from the HSQC plots in Fig. 6, the position of the hetero-heads before ultrasonication corresponds to 4.83/99.33 as the hetero-head H_1_/C_1_ signals of the main chain α-1,3-Man, and 4.35/103.21 as the hetero-heads H_1_/C_1_ signals of the branched chain β-1,6-Glc, and the hetero-heads region (90–110 ppm) in the two-dimensional NMR HSQCs after ultrasonication has a more In the 2D NMR HSQC after ultrasound treatment, the heterohead region (90–110 ppm) showed a more obvious change, in which the newly added heterohead H_1_/C_1_ at 4.97/92.82 ppm was the new heterohead signal formed by the glycosidic bond breaking after ultrasound treatment, which confirms the analysis of the corresponding structural changes in the carbon spectra. The comparison of the signals in the other regions showed no obvious changes, and it was hypothesized that the main structure of the mannan did not undergo any obvious changes. As can be seen from the COSY plot in Fig. 7, the hetero-head position before sonication corresponds to 4.83/3.63 as the correlation signals of the hetero-head H_1_/H_2_ of the main-chain α-1,3-Glc, and 4.35/3.35 as the signals of the hetero-head H_1_/H_2_ of the branched-chain β-1,6-Glc, and the hetero-head region (90–110 ppm) in the two-dimensional NMR HSQC after sonication has a more. There are obvious changes in the hetero-capsule region (90–110 ppm) after ultrasonication, in which the newly added hetero-capsule H_1_/H_2_ at 4.97/3.77 ppm are newly formed hetero-capsule signals broken after ultrasonication, confirming the analysis of the structural changes corresponding to the carbon spectra. Taken together, it is hypothesized that AAP changes before and after ultrasonic degradation to more branched 1,6-Glc glycosidic bond breakage. The clearer NMR spectra after sonication indicate improved solubility and structural uniformity of AAP, which can enhance its functional performance in various applications. The breaking of glycosidic bonds and the formation of new reducing ends suggest that ultrasonic treatment not only reduces molecular mass but also alters the branching structure of AAP, potentially making it more reactive. These structural modifications could lead to improved interaction with biological targets, such as enzymes or cellular receptors, thereby enhancing its physiological activity. The partial breakage of the β-1,6-Glc glycosidic bonds also implies that ultrasonic treatment can be used to selectively modify the polysaccharide structure, which could be advantageous for tailoring its properties for specific functional applications (Tang et al., 2024).

### 3.6 Solubility comparison

**Fig. 8:**
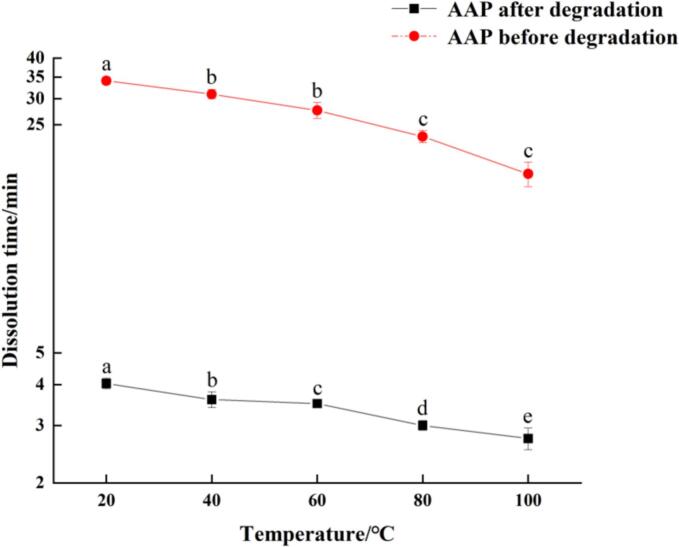
Solubility of polysaccharides.

The solubility of polysaccharides was determined by the time required for complete dissolution of polysaccharide samples at different temperatures, and the results are shown in Fig. 8. The results showed that the dissolution time of the predegraded AAP decreased significantly with the increase of temperature, while the degraded AAP remained unchanged. At 20 °C, the dissolution time of the predegraded AAP was approximately 9 times that of the post-degraded AAP. After the degradation treatment, the molecular mass of the polysaccharide decreased leading to a marked reduction in dissolution time (*P* < 0.01). Degraded AAP exhibited rapid solubility in water, which aligns with the NMR pressurized water peak in Fig. 4 above. Combined with the infrared spectra and NMR spectra, it can be seen that during the degradation process, ultrasonic treatment destroys the hydrogen bonds between the chains of polysaccharide molecules, leading to an increase in the number of short polymerized chains as well as an enhanced hydration tendency, which makes it easier to be dissolved and helps the polysaccharide to exert its physiological activity. Ultrasonic degradation alters the spatial configuration and connectivity of polysaccharides by breaking hydrogen bonds to a certain extent, which ultimately improves solubility. The significant improvement in solubility after ultrasonic degradation is a critical factor for the practical application of AAP in the food and pharmaceutical industries. Enhanced solubility not only facilitates easier incorporation into aqueous formulations but also improves the bioavailability of AAP when consumed. The breaking of hydrogen bonds and the formation of shorter polymer chains increase the hydration capacity of AAP, which is beneficial for its use as a thickening or stabilizing agent in food products. The improved solubility also suggests that ultrasonic treatment could be a viable method for overcoming the limitations of high molecular mass polysaccharides, which often suffer from poor solubility and limited physiological activity (Xiong et al., 2019).

### 3.7 Comparative analysis of antioxidant activity

#### 3.7.1 Comparison of DPPH free radical scavenging rate

**Fig. 9:**
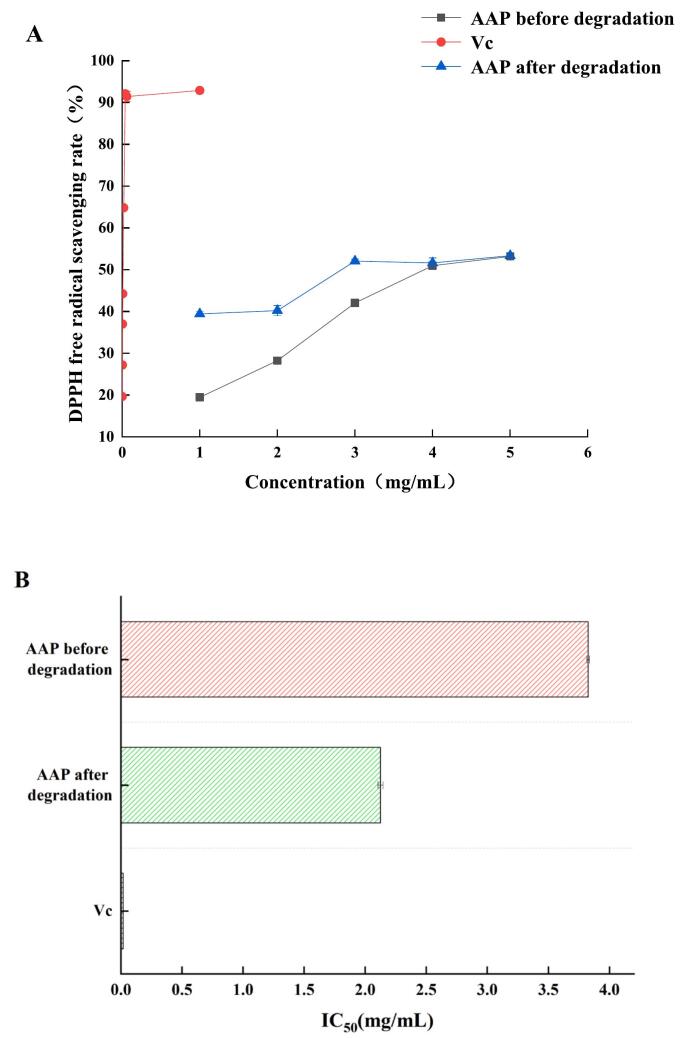
DPPH radical scavenging rate of AAP before and after degradation.

As shown in Fig. 9, APP before and after degradation showed different DPPH radical scavenging rates and the degraded AAP had a better antioxidant effect. The improved scavenging activity can be attributed to the structural changes in APP following ultrasonic degradation, which reduced its molecular mass and increased the exposure of hydroxyl groups, thus enhancing its DPPH radical scavenging activity (Chen et al., 2023). The IC_50_ of AAP for DPPH radical scavenging decreased after degradation, but it was much larger than that of Vc, and the specific values were as follows: IC_50_ (Vc) = 0.0876 mg/mL < IC_50_ (degraded AAP) = 2.066 mg/mL < IC_50_ (pre-degraded AAP) = 3.577 mg/mL. The enhanced DPPH radical scavenging activity after ultrasonic degradation highlights the effectiveness of this treatment in improving the antioxidant potential of AAP. The reduction in molecular mass and increased exposure of hydroxyl groups are likely responsible for the improved ability to donate hydrogen atoms, which is crucial for neutralizing free radicals. Although the IC_50_ value of degraded AAP is still lower than that of vitamin C, the significant improvement compared to pre-degraded AAP indicates that ultrasonic treatment can be an effective method to enhance the natural antioxidant properties of polysaccharides. This suggests potential applications of ultrasonically treated AAP as a natural antioxidant in functional foods and nutraceuticals (Lee et al., 2023).

#### 3.7.2 Comparison of hydroxyl radical scavenging rate

**Fig. 10:**
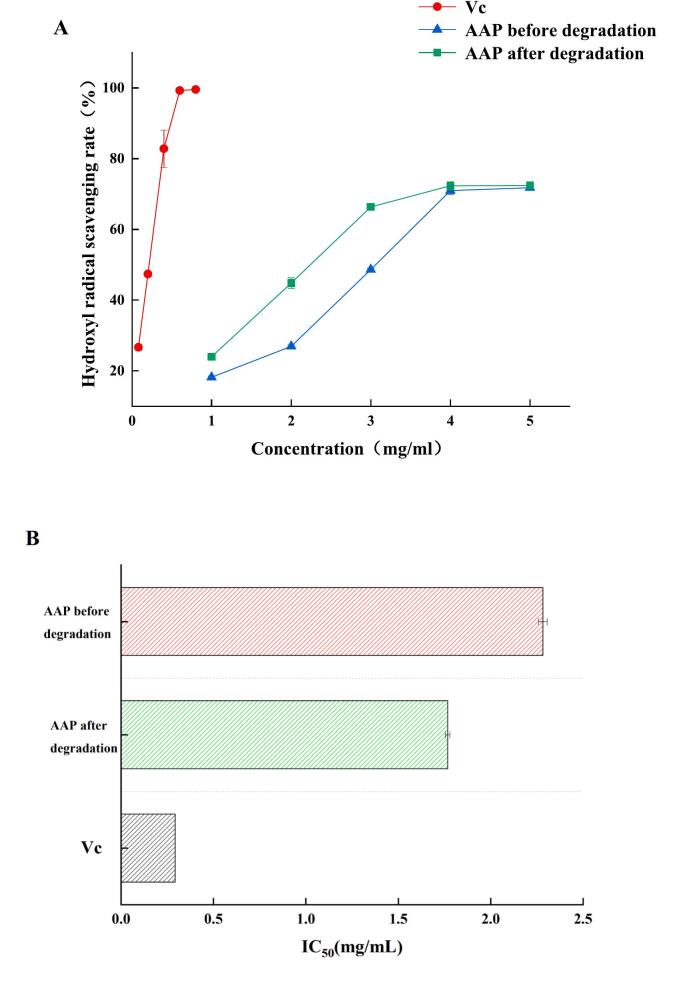
Hydroxyl radical scavenging rate of AAP before and after degradation.

As shown in Fig. 10, the hydroxyl radical scavenging rate of AAP before and after degradation was also significantly different, which was consistent with the experimental results of the DPPH radical scavenging rate. Ultrasonic degradation caused AAP to expose more hydroxyl groups, leading to an increase in hydroxyl radical scavenging capacity. The IC_50_ of degraded AAP for hydroxyl radical scavenging decreased but was greater than that of Vc, with the following values: IC_50_ (Vc) = 0.2932 mg/mL < IC_50_ (degraded AAP) = 1.768 mg/mL < IC_50_ (pre-degraded AAP) = 2.283 mg/mL. The increase in hydroxyl radical scavenging activity further supports the conclusion that ultrasonic degradation enhances the antioxidant properties of AAP. Hydroxyl radicals are highly reactive species that can cause significant damage to biological molecules, and the ability of AAP to scavenge these radicals is an important indicator of its antioxidant potential. The improved scavenging capacity after ultrasonic treatment suggests that the modified AAP could be more effective in protecting against oxidative stress, which is linked to various chronic diseases. This makes ultrasonically treated AAP a promising candidate for use in health supplements aimed at combating oxidative damage.

#### 3.7.3 Comparison of superoxide anion removal rate

**Fig. 11:**
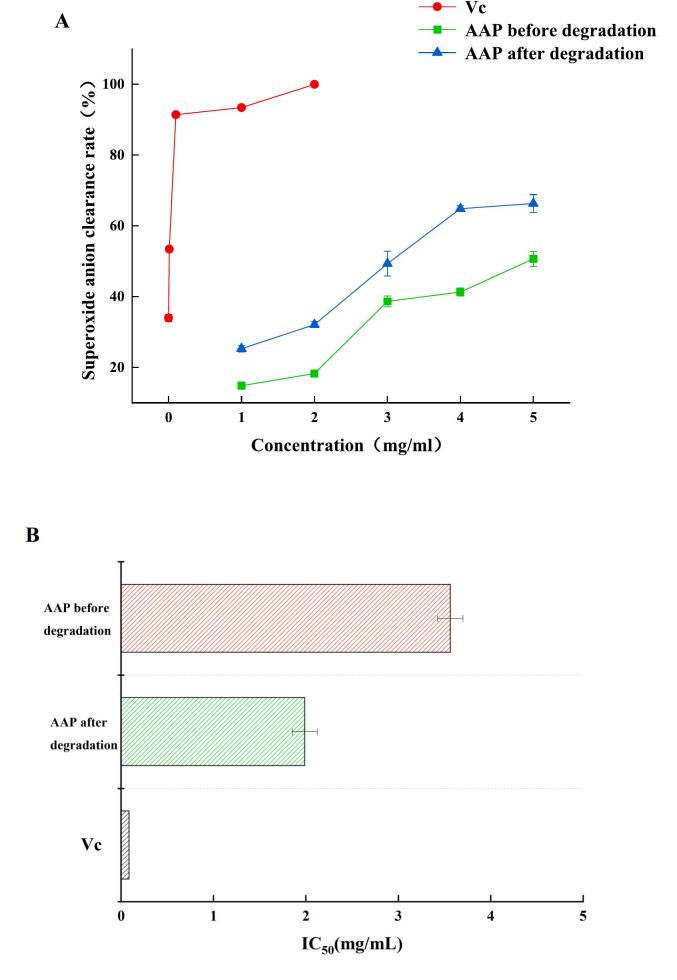
Superoxide anion scavenging rate of AAP before and after degradation.

As shown in Fig. 11, the superoxide anion scavenging rate of AAP before and after degradation was also significantly different, which is consistent with the above experimental results. Ultrasonic degradation caused AAP to expose more hydroxyl groups, leading to an increase in the superoxide anion scavenging capacity. The IC_50_ of degraded AAP for superoxide anion scavenging decreased but was greater than that of Vc, with the following values: IC_50_ (Vc) = 0.993 mg/mL < IC_50_ (degraded AAP) = 2.126 mg/mL < IC_50_ (pre-degraded AAP) = 3.835 mg/mL. The enhanced superoxide anion scavenging activity observed after ultrasonic degradation indicates that the treatment effectively improves the overall antioxidant profile of AAP. Superoxide anions are precursors to other reactive oxygen species, and their removal is crucial for preventing oxidative chain reactions. The increased scavenging ability suggests that ultrasonically treated AAP could serve as an effective natural antioxidant, protecting against oxidative stress in food products and biological systems. The consistent improvement across different radical types (DPPH, hydroxyl, and superoxide) demonstrates the broad-spectrum antioxidant potential of the treated AAP, making it a versatile ingredient for functional food applications (Yang et al., 2019).

The antioxidant effect of polysaccharides depends to some extent on the number of hydroxyl groups in contact with the outside world. Huang et al. used ultrasonic irradiation of the polysaccharides of *Helicoverpa armigera* and the number of hydrogen bonds of the polysaccharides increased after the treatment while the antioxidant activity of the polysaccharides was improved (Huang et al., 2020). Mtetwa et al. demonstrated that ultrasonic-assisted extraction of Cuspidata polysaccharides resulted in a higher number of hydrogen bonds and enhanced antioxidant activity compared to polysaccharides extracted using hot water (Mtetwa et al., 2020). All these findings are in agreement with the experimental results that ultrasonic degradation can significantly improve the antioxidant activity of *Bambusa nigra* polysaccharides.

### 3.8 Comparative analysis of in vitro hypoglycemic activity

**Fig. 12:**
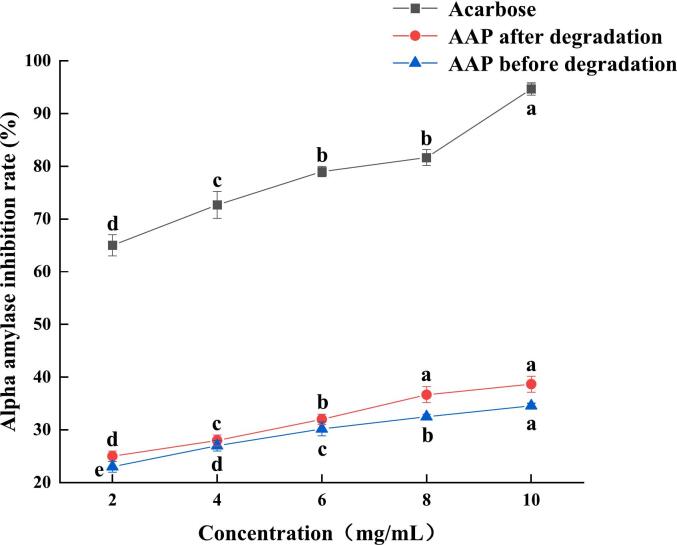
Effects of AAP before and after degradation on α- Amylase inhibition rate.

**Fig. 13:**
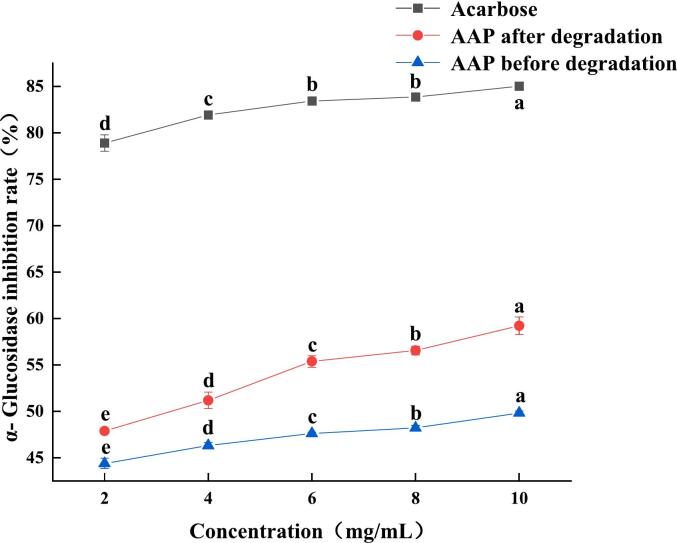
Effects of AAP before and after degradation on α- Glucosidase inhibition rate.

α-Amylase can break down carbohydrates such as starch into oligosaccharides, while α-glucosidase catalyzes the hydrolysis of glycosidic bonds and hydrolyzes oligosaccharides into glucose, leading to an increase in blood glucose levels. Therefore, inhibiting α-amylase as well as α-glucosidase activity can effectively delay the release of glucose, lowering postprandial blood glucose levels and providing a hypoglycemic effect (Tang et al., 2023). As shown in Fig. 12, the inhibition rates of AAP before and after ultrasonic degradation were 34.08 % and 39.14 %, respectively, at the mass concentration of 10 mg/mL, and the inhibition rate was increased by 5.06 % with the IC_50_s of 19.33 and 14.5 mg/mL, respectively, which indicated that ultrasonic degradation of AAP could inhibit the decomposition of high glucose by inhibiting the activity of α-amylase, and thus achieve the purpose of hypoglycemia. The inhibition of these enzymes helps regulate glucose release, thereby achieving a hypoglycemic effect (Wang et al., 2017). As shown in Fig. 13, the inhibition rate of α-glucosidase increased with the increase in mass concentration. At a mass concentration of 10 mg/mL, the inhibition rates of AAP before and after ultrasonic degradation were 52.43 % and 58.24 %, respectively, and the inhibition rate of α-glucosidase increased by 5.81 %, and the IC_50_s of the two were 5.703 and 2.076 mg/mL, respectively. The improved inhibition rates of both α-amylase and α-glucosidase after ultrasonic degradation suggest that the treated AAP has enhanced potential as a hypoglycemic agent. By inhibiting these enzymes, AAP can effectively reduce the rate of glucose release from carbohydrates, which is beneficial for managing blood glucose levels. The reduction in IC_50_ values indicates that a lower concentration of the degraded AAP is required to achieve the same inhibitory effect, demonstrating increased efficacy. This makes ultrasonically treated AAP a promising natural ingredient for developing functional foods or supplements aimed at glycemic control, particularly for individuals with diabetes or those at risk of developing diabetes (Wang et al., 2023b).

### 3.9 Comparative analysis of in vitro hypolipidemic activity

**Fig. 14:**
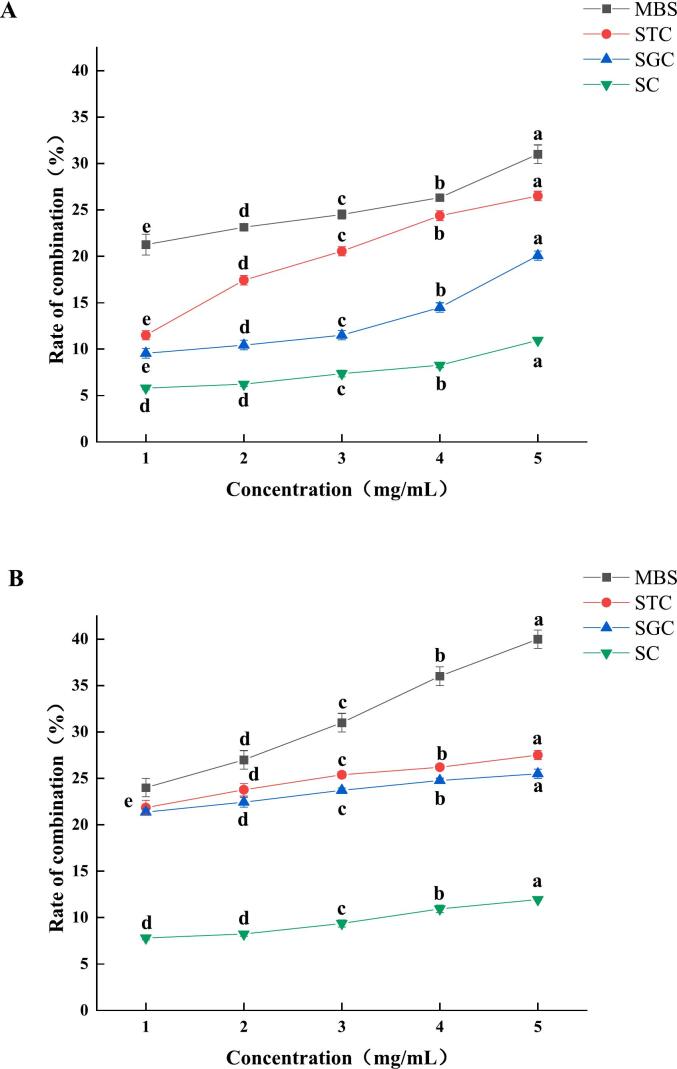
Binding rates of AAP to SC, STC, SGC, and MBS before (above) and after (below) degradation. MBS(Mixed Bile Salts), STC (Sodium Taurocholate), SGC(Sodium Glycocholate), SC(Sodium Cholate).

Bile acids generally exist in the body in the form of salts and bound forms and are excreted in the form of bile acids. AAP adsorbs bile acid salts through the viscous network, promoting bile acids excretion and accelerating the conversion of cholesterol to bile acids, thereby reducing cholesterol levels and achieving a lipid-lowering effect. As shown in Fig. 14, the binding capacity of AAP for bile acid salts increased significantly with the increase of polysaccharide concentration, but there were differences in the binding capacity for different bile acid salts, and the ultrasonically degraded AAP had a better adsorption capacity for bile acid salts. The binding capacity of AAP for the four bile acid salts, in descending order, was as follows: MBS (Mixed Bile Salts) > STC (Sodium Taurocholate) > SGC (Sodium Glycocholate) > SC (Sodium Cholate). The binding rate was 32.34 ± 3.0 % for AAP at the concentration of 5 mg/mL. The binding rates of AAP at a concentration of 5 mg/mL were 32.34 ± 1.12 %, 26.52 ± 0.54 %, 20.6 ± 0.51 %, and 10.98 ± 0.26 %, respectively. The binding rates of degraded AAP were 41.57 ± 0.87 %, 27.52 ± 0.68 %, 25.5 ± 0.26 %, and 11.89 ± 0.41 % at a concentration of 5 mg/mL, respectively. This indicates that AAP has a certain binding ability to different bile acid salts, among which the binding effect on MBS is the most obvious, and ultrasound can promote the binding of AAP to bile acid salts. Bile acid salts in vivo play an important role in lowering blood lipids and cholesterol, and the bile acid salt binding rate of polysaccharides may be related to their complex spatial structure. The results indicated that AAP could enhance hypolipidemic effects by binding to bile salts, and ultrasonic degradation further increased this binding capacity. Ultrasonically treated AAP promoted bile acid excretion, stimulated cholesterol conversion into bile acids and reduced blood cholesterol levels. The improved binding ability is likely due to increased surface area and exposed functional groups, making ultrasonically treated AAP a promising ingredient for functional foods or dietary supplements targeting cholesterol management and cardiovascular health. Additionally, its ability to bind bile acids may support gut health by modulating bile acid metabolism (Pan et al., 2017).

## 4 Conclusion

Ultrasonic degradation effectively enhanced the functional properties of AAP by significantly reducing its molecular mass, improving solubility, and modifying its structural characteristics. These changes led to notable improvements in its antioxidant, hypoglycemic, and hypolipidemic activities, including increased hydroxyl group exposure that enhanced free radical scavenging, as well as improved α-amylase and α-glucosidase inhibition rates, which support its potential for blood glucose regulation. Furthermore, the enhanced bile acid binding capacity underscores AAP's potential role in cholesterol metabolism. This study demonstrates that ultrasonic degradation is a green and efficient approach to improving AAP's bioactive properties, making it a promising candidate for applications in functional foods and health supplements. Future research should focus on optimizing ultrasonic treatment parameters and conducting in vivo studies to better understand the underlying mechanisms and confirm its health benefits in real biological systems.

## CRediT authorship contribution statement

**Te Yu:** Software, Methodology, Conceptualization. **Yonggang Dai:** Writing – original draft, Data curation. **Zifei Wang:** Visualization, Investigation. **Yang Gao:** Supervision. **Qiong Wu:** Writing – review & editing, Project administration, Funding acquisition.

## Declaration of competing interest

The authors declare that they have no known competing financial interests or personal relationships that could have appeared to influence the work reported in this paper.

## Data Availability

The authors do not have permission to share data.
